# Cross-Adversarial Learning for Molecular Generation in Drug Design

**DOI:** 10.3389/fphar.2021.827606

**Published:** 2022-01-21

**Authors:** Banghua Wu, Linjie Li, Yue Cui, Kai Zheng

**Affiliations:** ^1^ School of Cyber Science and Engineering, Sichuan University, Chengdu, China; ^2^ School of Computer Science and Engineering, University of Electronic Science and Technology of China, Chengdu, China

**Keywords:** molecular generation, adversarial learning, projected gradient descent, adversarially regularized autoencoder, generative adversarial network

## Abstract

Molecular generation is an important but challenging task in drug design, as it requires optimization of chemical compound structures as well as many complex properties. Most of the existing methods use deep learning models to generate molecular representations. However, these methods are faced with the problems of generation validity and semantic information of labels. Considering these challenges, we propose a cross-adversarial learning method for molecular generation, CRAG for short, which integrates both the facticity of VAE-based methods and the diversity of GAN-based methods to further exploit the complex properties of Molecules. To be specific, an adversarially regularized encoder-decoder is used to transform molecules from simplified molecular input linear entry specification (SMILES) into discrete variables. Then, the discrete variables are trained to predict property and generate adversarial samples through projected gradient descent with corresponding labels. Our CRAG is trained using an adversarial pattern. Extensive experiments on two widely used benchmarks have demonstrated the effectiveness of our proposed method on a wide spectrum of metrics. We also utilize a novel metric named Novel/Sample to measure the overall generation effectiveness of models. Therefore, CRAG is promising for AI-based molecular design in various chemical applications.

## 1 Introduction

The primary goal of the drug design process is to find new chemical compound structures that can adjust the given protein activities in a desired way. This process takes about 10 years and is accompanied by a huge expenditure of funds. Generally, a new drug needs to go through four stages before putting into the market: drug discovery, pre-clinical research, clinical research, and approval of listing (Khan et al., 2021). De novo drug design (Hartenfeller and Schneider, 2010) through existing computer technology can speed up drug development and save research costs. The tasks involved in *de novo* drug design include molecular generation (Gómez-Bombarelli et al., 2016; Cao and Kipf, 2018; Jin et al., 2018; You et al., 2018; Madhawa et al., 2019; Popova et al., 2019; Zhang et al., 2019; Hong et al., 2020; Zang and Wang, 2020; Bagal et al., 2021), drug and drug interactions (DDI) (Li et al., 2021; Lin et al., 2021; Lyu et al., 2021; Zhao et al., 2021), disease associations (Ding et al., 2020; Lei et al., 2020; Lei and Zhang, 2020; Mudiyanselage et al., 2020; Lei X.-J. et al., 2021; Lei X. et al., 2021; Wang Y. et al., 2021; Lei and Zhang, 2021; Yang and Lei, 2021; Zhang et al., 2021), and so on. Traditional molecular generation tasks follow a two-step strategy to design new molecules: synthesizing alternative compounds clinically and conducting experiments. However, these methods are faced with two huge challenges. One is that the chemical molecule space is discrete and vast, which has been estimated to be between 10^23^ and 10^60^ (Polishchuk et al., 2013). The other is that the relation between molecule structure and properties is quite sensitive, even small structural changes will lead to significant molecule property variations.

**FIGURE 1:**
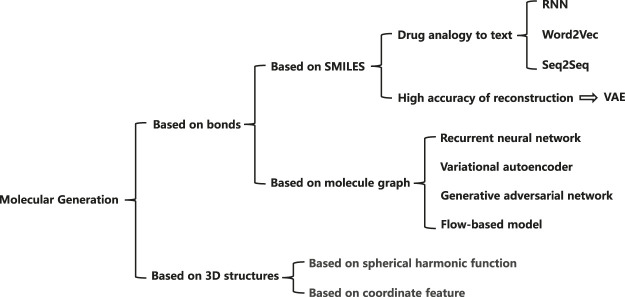
Current work on molecular generation for drug representation.

In the past few years, with the development of deep learning techniques, deep learning based methods have been proposed to overcome the problems in previous methods. Most of the existing methods learn to represent the molecular characteristics using deep networks and construct the relationship between atoms to express atomic information. Figure 1 illustrates the mainstream molecular generation methods, which can be summarized into two directions, i.e., bond-based methods and 3D structure-based methods. Among the bond-based methods, simplified molecular input linear entry specification (SMILES) (Merkwirth and Lengauer, 2005) and molecular graph (Simonovsky and Komodakis, 2018) are two widely used methods. The SMILES usually regards drug as a sequence with rich semantics. Most of the SMILES methods extract molecular information through recurrent neural networks (Segler et al., 2017), word2vec (Jaeger et al., 2018), seq2seq (Xu et al., 2017), or other language models. Besides, variational auto-encoders (VAEs) can achieve high reconstruction accuracy, which is of great significance for molecular generation tasks. Rafael et al. (Gómez-Bombarelli et al., 2016) propose to convert discrete representations of molecules into multi-dimensional continuous ones. Molecular graph based methods generally express atomic information through a node tensor, and the relation between atoms through an adjacency matrix to retain more molecular information. Researchers generally process molecular graphs according to four technical routes, based on the RNN model (Popova et al., 2019), based on the VAE model (Simonovsky and Komodakis, 2018), based on the GAN model (Cao and Kipf, 2018; Wang F. et al., 2021), and based on the flow model (Zang and Wang, 2020; Ma and Zhang, 2021). Among them, the methods based on the VAE model and the GAN model both show excellent effects. At present, researchers mainly advance in-depth research on bond-based generation models. In order to improve the effect of the generation models, researchers also mine the 3D structure information of molecules. The current methods are mainly based on spherical harmonic function (Tanaka et al., 1993) and based on coordinate feature (Guu et al., 2015).

However, both the VAE-based and GAN-based methods mentioned above still remain some problems. Firstly, the majority of the VAE-based models utilize Kullback-Leibler divergence to approximately calculate the distribution of the source sampling space and the target space. This will cause the generation distribution of the final generative model can be greatly different from the actual distribution. Because the exact characteristics of the target distribution are unknown, VAE-based methods may result in unnatural molecules and reduce the validity of generated molecules. Secondly, the GAN-based model only trains a generator and a discriminator, which makes the representation of the latent vector unknown and the model difficult to be controlled. Thirdly, most GAN-based methods can not make good use of data labels and waste the properties information of the molecules.

To address the above issues, in this paper, we propose a cross-adversarial learning method for molecular generation taking the advantages of both VAE-based and GAN-based methods, namely CRAG. On one hand, an adversarially regularized autoencoder (Zhao et al., 2018) is combined with a discrete autoencoder to generate the GAN-regularized latent representation, which utilizes a more flexible prior distribution to provide a smoother discrete coding space. On the other hand, we utilize an adversarial method of projected gradient descent (Madry et al., 2018) to generate adversarial samples, which achieves data augmentation without changing the real molecular distribution and solves the problem of estimating the representation distribution. The method of training CRAG based on GAN-like structure through adversarial samples is called cross-adversarial learning. Therefore, our model can achieve high validity and uniqueness in molecular generation.

In general, the contributions of this paper can be summarized as follows:

• We propose an adversarially regularized encoder-decoder based on projected gradient descent to generate discrete variables, namely CRAG. CRAG combines the advantages of both the VAE-based and GAN-based methods, which can provide a smoother discrete coding space.
• We utilize an adversarial method of projected gradient descent to generate adversarial samples with property information, which augmented data without changing the real molecular distribution and solves the problem of estimating the representation distribution. This generation process through adversarial training achieves cross-adversarial training of the CRAG.
• The effectiveness of the proposed approach is analyzed and confirmed through extensive experiments on two public datasets. The results show that CRAG outperforms state-of-the-art models in terms of the validity, uniqueness, and novelty of each generated molecule.

## 2 Related Work

### 2.1 Molecular Generation

Molecular generation is a part of *de novo* drug design. The generative model can capture potential rules for data distribution, so that the model can infer the molecules through the reverse mapping between the structure and the properties under the constraints of the given property conditions. The generative model needs to have high reconstruction accuracy, and the model should make the generated new molecules have high validity, high uniqueness, and high novelty.

We can classify the widely used molecular generation models based on the methods of learning data distribution and the way of generating molecules. According to the idea of “drug analogy to text”, DeepGMG (Li et al.,2018) and MolecularRNN (Popova et al.,2019) can analyze molecular information by constructing recurrent neural networks, which is in a step-by-step fashion by adding nodes and edges one by one. But the reconstruction accuracy of these models is very low. Therefore, researchers utilize the high reconstruction accuracy of VAE-based models, which assume a simple variational distribution of the latent vectors. Such as GraphVAE (Simonovsky and Komodakis, 2018), which generates a molecular in a one-shot fashion by a single step. JT-VAE (Jin et al., 2018) also presents good performance on molecular generation by using junction trees. In order to solve the problem that it is difficult for VAE-based models to estimate the distribution, GAN-based models such as MolGAN (Cao and Kipf, 2018) and GCPN (You et al., 2018) have appeared. Of course, for the purpose of taking advantages of both VAEs and GANs, the ARAE (Hong et al., 2020) method is a wise choice. Flow-based model is another major type of generative model method besides the above methods. For example, the more well-known are GraphNVP (Madhawa et al., 2019) and MoFlow (Zang and Wang, 2020). Details are shown in Table 1.

**TABLE 1:** Mainstream methods of molecular generation.

Method	RNN	VAE	GAN	Flow	One-shot	Sequential
DeepGMG Li et al. (2018)	*✓*	—	—	—	—	*✓*
MolecularRNN Popova et al. (2019)	*✓*	—	—	—	—	*✓*
GraphVAE Simonovsky and Komodakis. (2018)	—	*✓*	—	—	*✓*	—
JT-VAE Jin et al. (2018)	—	*✓*	—	—	—	*✓*
MolGAN Cao and Kipf. (2018)	—	—	*✓*	—	*✓*	—
GCPN You et al. (2018)	—	—	*✓*	—	—	*✓*
ARAEHong et al. (2020)	—	*✓*	*✓*		*✓*	—
GraphNVP Madhawa et al. (2019)	—	—	—	*✓*	*✓*	—
MoFlow Zang and Wang. (2020)	—	—	—	*✓*	*✓*	—
**Ours**	—	*✓*	*✓*	—	*✓*	—

### 2.2 Adversarially Regularized Autoencoder

Adversarially Regularized Autoencoder (ARAE) (Zhao et al., 2018) was proposed to address the aforementioned problem of VAE-based and GAN-based methods. It combines a discrete autoencoder with a GAN-regularized latent representation, which utilizes a more flexible prior distribution to provide a smoother discrete coding space (Kong and Kim, 2019). The encoder network parameterized by *θ* outputs the true latent vector *z* from the given input *x*. The decoder network parameterized by *ϕ* reconstructs the inputs from the latent vector. According to the idea of GANs, the generator parameterized by *ψ* outputs the distribution of generated random vector $\hat{z}$ . Thus, adversarially regularized autoencoder proposes an objective function as:

$$\underset{\theta ,\phi ,\psi }{\min}E_{x∼p_{r}\left(x\right)}\left[L_{rec}\left(\theta ,\phi \right)+W\left(p_{\theta }\left(z\right),p_{\psi }\left(\hat{z}\right)\right)\right],$$ (1)

where the reconstruction loss caused by encoder and decoder can be written as:

$$L_{rec}\left(\theta ,\phi \right)=E_{z∼p_{\theta }\left(z\right)}\left[-\log p_{\phi }\left(z|x\right)\right],$$ (2)

and *W* is the Wasserstein distance (Arjovsky et al., 2017) between *p*_*θ*_, the distribution from a discrete encoder model, and *p*_*ψ*_, a prior distribution. The *W* function which is adversarially optimized for the generator and encoder, can be written as:

$$W\left(p_{\theta }\left(z\right),p_{\psi }\left(\hat{z}\right)\right)=\underset{\omega }{\max}E_{z∼p_{\theta }\left(z\right)}\left[f_{\omega }\left(z\right)\right]-E_{\hat{z}∼p_{\psi }\left(\hat{z}\right)}\left[f_{\omega }\left(\hat{z}\right)\right],$$ (3)

with the 1-Lipschtiz continuity $\left\|f_{\omega }\right\|\leq 1$ . After training, the distributions of *p*_*θ*_ and *p*_*ϕ*_ become identical, and we can also use these generated samples as the input of the decoder to generate new molecules.

### 2.3 Projected Gradient Descent

Projected gradient descent is a first-order attack method (Madry et al., 2018), which demonstrates excellent adversarial robustness. Adversarial training through the projected gradient descent can effectively improve the robustness of the model. The model is divided into two parts, which are the maximization of the internal loss function and the minimization of external experience risk. For each data point *x*, we introduce a set of perturbations S that formalizes the manipulative power of the adversary. Considering underlying the case of a standard classification task, whose basic data distribution D is on the paired example *x* and the corresponding label *y*. Assume that a suitable loss function L(θ,x,y) is obtained. *θ* is the set of classification model parameters. Our goal is to find *θ* that minimizes the risk $E_{\left(x,y\right)∼D}$ :

$$\rho \left(\theta \right)=E_{\left(x,y\right)∼D}\left[\underset{\delta \in S}{\max}L\left(\theta ,x+\delta ,y\right)\right].$$ (4)

Equation 4 shows the implementation details of the projected gradient descent for our CRAG. The goal of inner maximization is to find the corresponding adversarial samples in the original data, so that it can achieve high loss. The goal of outer minimization is to find suitable network parameters to train a more robust neural network to defend against attacks samples.

## 3 Methods

**FIGURE 2:**
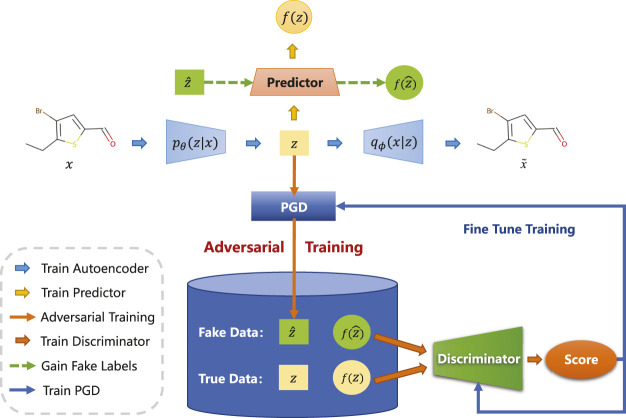
Illustration of the overall architecture. CRAG consists of an adversarially regularized encoder-decoder block, a property predictor block, and a projected gradient descent block. In particular, *p*_*θ*_(*z*|*x*) and *q*_*ϕ*_(*x*|*z*) denote the probabilistic encoder and the probabilistic decoder. The property prediction network *f*_*ω*_ is used to predict the property of molecules. Projected gradient descent block generates adversarial samples $\hat{z}$ and labels $f\left(\hat{z}\right)$ . The discriminator is only used in the training process to enforce regularization.

In this section, we will elaborate on the details of the proposed cross-adversarial learning method. As shown in Figure 2, CRAG mainly consists of the following components: an encoder and a decoder are used to learn latent representation, a property predictor that is used to predict molecular properties, a projected gradient descent block for generating adversarial samples, and a discriminator that is used to judge whether the input molecule is a true or adversarial sample.

### 3.1 Adversarially Regularized Encoder-Decoder Block

Adversarially regularized encoder-decoder block combines a discrete autoencoder with a GAN-regularized latent representation (Zhao et al., 2018). It is trained to optimize three parts of the model (Kong and Kim, 2019). The first part of the adversarially regularized encoder-decoder is a variational autoencoder, which minimizes the reconstruction loss to obtain a continuous latent representation of a molecule. The second part is a discriminator, which maximizes the distance between real data distribution and fake data distribution to achieve an adversarial attack. The third part is the encoder and generator, which minimizes the distance between real data distribution and fake data distribution to generate adversarial samples.

#### 3.1.1 Autoencoder

Define $x\in R^{d}$ to be a set of molecules where *d* presents molecular features. The encoder network *p*_*θ*_(*z*|*x*) aims to convert the given input data *x* into a latent vector *z*. The input data *x* of the model are the SMILES representation of the molecular structure, which is a discrete random variable. The decoder network *q*_*ϕ*_(*x*|*z*) aims to convert the latent vector *z* into molecule $\tilde{x}$ . According to Eq. 5, we can obtain $\tilde{x}$ by argmax function.

$$\tilde{x}=\arg\underset{x}{\max}q_{\phi }\left(x|z\right)$$ (5)

The parameters are trained based on the cross-entropy reconstruction loss:

$$L_{rec}\left(\theta ,\phi \right)={\left\|x-\tilde{x}\right\|}^{2}.$$ (6)

Our goal is to minimize the reconstruction loss of the autoencoder. Thus, we can not only find a suitable drug molecule representation but also help to find the specific structure of the molecule in the *de novo* drug design.

#### 3.1.2 Generative Adversarial Networks

GANs are a class of parameterized implicit generative models (Goodfellow et al., 2014). GAN-based methods mainly focus on two optimization goals: one is the problem of maximizing the distribution distance of the discriminator, and the other is the problem of minimizing the distribution distance between encoder and generator. This optimization process can be regarded as a dynamic game between the discriminator and the generator.

For the first optimization, the goal of the discriminator is to distinguish the generated samples from the real data as much as possible, which can be written as:

$$\underset{\omega \in W}{\min}L_{cri}\left(\omega \right)=\underset{\omega \in W}{\max}E_{z∼p_{\theta }}\left[f_{\omega }\left(z\right)\right]-E_{\hat{z}∼g_{\psi }}\left[f_{\omega }\left(\hat{z}\right)\right],$$ (7)

where *f*_*ω*_ is the property prediction network parameterized by *ω*. During the training process, the generating samples $\hat{z}$ are used to train the discriminator *g*_*ψ*_. The discriminator generates a score for each input and performs gradient backpropagation through the loss function composed of the score and the label.

For the second optimization, The goal of the generator *g*_*ψ*_ is to generate real samples to deceive the discriminator as much as possible. We introduce a property predictor block to replace the traditional generator. In the traditional adversarially regularized autoencoder structure, a generator takes latent vector $\hat{z}$ sampled from a noise distribution N∼(0,1) . Through training, the gap between the generated sample and the real data is continuously narrowed to confuse the discriminator. Eq. 8 shows the optimization process.

$$\underset{\theta ,\psi }{\min}L_{gen}\left(\theta ,\psi \right)=\underset{\theta ,\psi }{\min}E_{z∼p_{\theta }}\left[f_{\omega }\left(z\right)\right]-E_{\hat{z}∼g_{\psi }}\left[f_{\omega }\left(\hat{z}\right)\right]$$ (8)

In this paper, we utilize a projected gradient descent block to generate adversarial samples with property information and achieve cross-adversarial of our CRAG. The projected gradient descent block will be presented in the next section.

#### 3.1.3 Property Predictor Block

In order to improve efficiency, the property predictor *f*_*ω*_ is implemented as a two-layer perceptron, which can effectively mine the real data distribution information. After the training of the predictor, the generated adversarial samples can be labeled to the corresponding adversarial labels by the predictor.

### 3.2 Projected Gradient Descent Block

**FIGURE 3:**
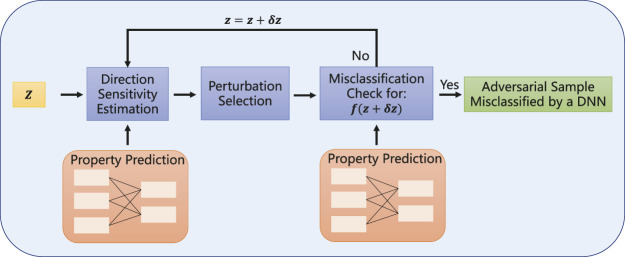
Details of the projected gradient descent block. Projected gradient descent continues to add small perturbations *δ* to the real sample *z* until it successfully interferes with their label categories, thereby generating adversarial sample $\hat{z}$ with property information.

Based on the adversarially regularized autoencoder, we use projected gradient descent to add property information for adversarial samples and achieve cross-adversarial learning for CRAG. Projected gradient descent is a gradient-based adversarial attack model (Madry et al., 2018). Through the previous introduction, the property predictor is parameterized by *ω*. For each latent vector *z*, we introduce a set of allowed perturbations $S\subseteq R^{d}$ , which can formalize the manipulative power of the adversary. With the gradient descent method, the perturbation is gradually added based on the original classification label, as shown in Figure 3. After calculating the gradient by the property predictor, the projected gradient descent block can generate adversarial samples for CRAG.

As a result, the adversarial sample $\hat{z}$ can be written as:

$$\hat{z}=\underset{z+S}{\prod }\left(z+\alpha \mathrm{sgn}\left(\nabla_{z}L\left(\omega ,z,y\right)\right)\right),$$ (9)

where L(ω,z,y) is the cross-entropy loss for the property predictor, and *α* is a set of steps.

### 3.3 Overall Model and Training

After introducing all the building blocks of our work, we give the final training objective and explain the optimization process, as shown in Algorithm 1. First, we trained an autoencoder to convert discrete data forms into continuous latent vectors. Then we put the obtained latent vector *z* into the PGD module to generate adversarial samples $\hat{z}$ . Based on the adversarial samples, we can alternatively train the discriminator and the generator with a GAN-like structure. This training process can be regarded as cross-adversarial training.

Algorithm 1 CRAG training.

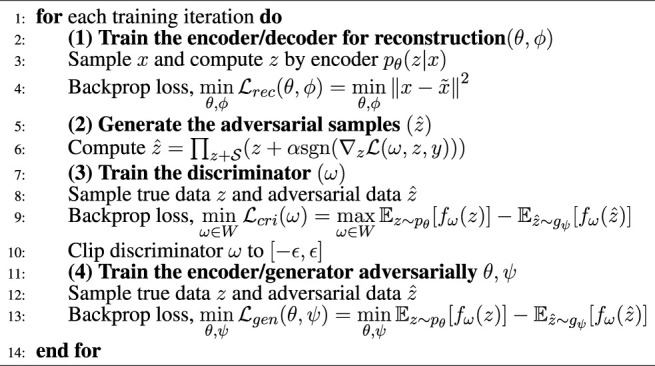

The training process of CRAG involves the optimization process of three objectives, as shown in Eq. 10. The first is to minimize the reconstruction loss of the variational autoencoder, the second is to maximize the distribution distance of the discriminator, and the third is to minimize the distribution distance between the encoder and the generator.

$$\left\{\begin{array}{cc} \underset{\theta ,\phi }{\min}L_{rec}\left(\theta ,\phi \right) & =\underset{\theta ,\phi }{\min}{\left\|x-\tilde{x}\right\|}^{2}\\ \underset{\omega \in W}{\min}L_{cri}\left(\omega \right) & =\underset{\omega \in W}{\max}E_{z∼p_{\theta }}\left[f_{\omega }\left(z\right)\right]-E_{\hat{z}∼g_{\psi }}\left[f_{\omega }\left(\hat{z}\right)\right]\\ \underset{\theta ,\psi }{\min}L_{gen}\left(\theta ,\psi \right) & =\underset{\theta ,\psi }{\min}E_{z∼p_{\theta }}\left[f_{\omega }\left(z\right)\right]-E_{\hat{z}∼g_{\psi }}\left[f_{\omega }\left(\hat{z}\right)\right] \end{array}\right.$$ (10)

## 4 Results

In this section, to verify the effectiveness of our method CRAG, we perform the experiences on two publicly available datasets (Polykovskiy et al., 2018) which are widely used for molecular generation.

### 4.1 Experimental Setup

#### 4.1.1 Evaluation Metrics

In the molecular generation task, three metrics of validity, uniqueness, and novelty are commonly used to evaluate the effect of the generative model. These three metrics are usually checked by using RDKit (Bento et al., 2020). In order to evaluate the comprehensive performance of the CRAG on these three metrics, we have introduced a new metric called Novel/Sample (Hong et al., 2020), which refers to the multiplication of the three metrics. Specifically, we utilize the following four metrics to evaluate the performance of our method.

• **Validity** refers to the ratio of the number of valid molecules to the number of generated samples.
• **Novelty** refers to the ratio of the number of molecules not included in the training set to the number of unique molecules.
• **Uniqueness** refers to the ratio of the number of unrepeated molecules to the number of valid molecules.
• **Novel/Sample** (Hong et al., 2020) refers to the ratio of the number of valid, unique, and novel molecules to the total number of generated samples.

#### 4.1.2 Evaluation Baselines

To demonstrate the effectiveness of CRAG, we compare CRAG with state-of-the-art molecular generation methods as follows.

• **ChemicalVAE (** Gómez-Bombarelli et al., 2016 **)** converts the SMILES representation of molecules to form a multidimensional continuous representation based on variational autoencoder, which is jointly trained on properties.
• **GrammarVAE** (Kusner et al., 2017) encodes and decodes directly to parse trees, which are represented as context-free grammar. GrammarVAE can effectively guarantee the validity of the generated outputs.
• **GraphVAE** (Simonovsky and Komodakis, 2018) is formulated in the framework of variational autoencoder, sidestep hurdles associated with linearization of discrete structures by having a decoder output a probabilistic fully connected graph of a predefined maximum size directly at once.
• **GraphVAE/imp** (Simonovsky and Komodakis, 2018) is implicit node probability based on the GraphVAE model, which assumes the independence of node and edge probabilities, and allows for isolated nodes or edges. Taking further advantage of the fact that the molecule is a connected graph, studied the effect of making node probabilities a function of edge probabilities.
• **GraphVAE NoGM** (Simonovsky and Komodakis, 2018) learns to reproduce particular node permutations in the training set based on the GraphVAE model. It investigates the importance of graph matching by using identity assignment instead, which corresponds to the canonical ordering of SMILES strings from RDKit.
• **MolGAN** (Cao and Kipf, 2018) uses Generative Adversarial Networks (GANs) to directly manipulate graph structure data and is combined with reinforcement learning objectives to encourage the generation of molecules with specific desired chemical properties.
• **ARAE** (Hong et al., 2020) basically uses latent variables like VAEs, but the distribution of the latent variables is obtained by adversarial training like GANs.

#### 4.1.3 Datasets

In order to train and test CRAG, we used QM9 and ZINC datasets, which are widely used in experiments and comparisons of various data-driven molecular property prediction methods (Irwin et al., 2012). The QM9 dataset (Ruddigkeit et al., 2012) contains about 133, 885 molecules of up to 9 heavy atoms: carbon (C), oxygen (O), nitrogen (N), fluorine (F), and so on. (Ramakrishnan et al., 2014). Among them, 10,000 molecules are selected as the test set. The ZINC dataset used in our experiments contains about 249, 455 molecules, which were randomly selected from the drug-like subset of the ZINC database. Data is split in the same way as the QM9 data set, and 10,000 molecules are also selected as the test set. The processing details of the ZINC database are consistent with ChemicalVAE (Gómez-Bombarelli et al., 2016).

#### 4.1.4 Implementation Details

Our whole architecture is optimized with Adam (Kingma and Ba, 2015) optimizer. Specifically, the initial learning rates of the autoencoder, generator, and discriminator are set as 10^–3^, 10^–5^, and 2 × 10^–6^, respectively. Each of the encoder and the decoder are composed of a single LSTM layer, and the dimension of outputs is 300. The LSTM layer of the encoder reads sequential SMILES strings and transforms them into latent vectors. For adversarial training, we use two fully-connected layers with a hidden dimension of 200 for the generator and the discriminator. The predictor network is also composed of two fully-connected layers with a hidden dimension of 200. Our model is implemented with PyTorch.

### 4.2 Experimental Results

#### 4.2.1 Smoother Discrete Coding Space

**FIGURE 4:**
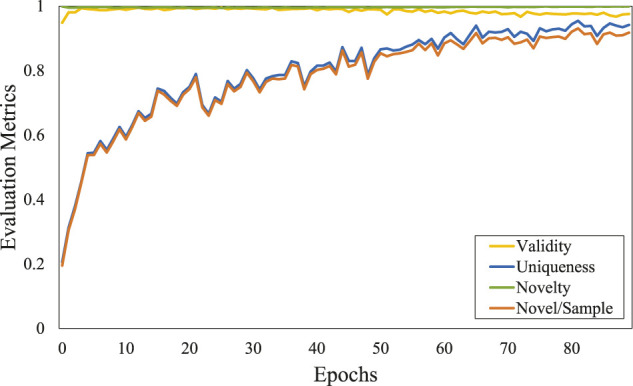
Convergence of the four evaluation metrics with the ZINC dataset.

It is well known that the training of generative adversarial networks (GANs) is relatively unstable. Here, we show the convergence of the four evaluation metrics for the first 80 epochs with the ZINC dataset in Figure 4.

**FIGURE 5:**
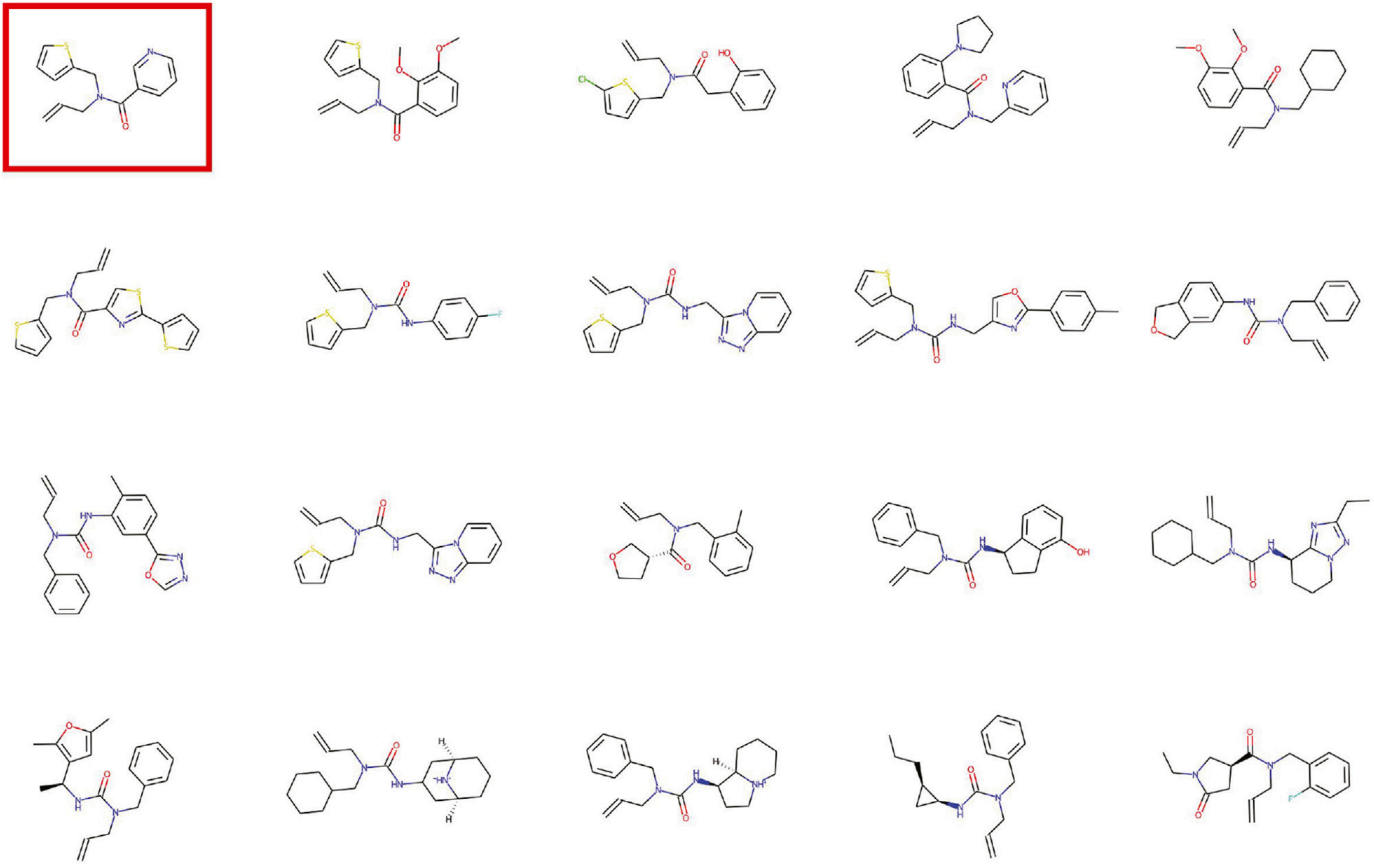
Visualization of the latent space for molecular generation by a given molecule. The red circled molecule is the given molecule.

In each epoch, 10, 000 molecules were generated and the four metrics were calculated. In the process of generating molecules, the PGD module is used to gradually increase the tiny noise to generate molecules with tiny changes. The research of the variation process of the generated molecules in the latent space is of great significance to the application of molecule generation. Figure 5 shows the visualization of the latent space for molecular generation by a given molecule.

#### 4.2.2 Performance of CRAG on Molecular Generation

We compare the performance of CRAG to those of ChemicalVAE, GrammarVAE, GraphVAE, MolGAN, and ARAE. All of these models are trained based on VAEs or GANs. We summarize the metrics of validity, uniqueness, and novelty, as shown in Table 2.

**TABLE 2:** Performance of benchmark models and our CRAG model on the QM9 and the ZINC datasets. Baseline results are taken from (Cao and Kipf, 2018), and Baseline results are based on the QM9 dataset.

Method	Validity (A)	Uniqueness (B)	Novelty (C)	Novel/Sample (AxBxC)
ChemicalVAE	0.103	0.675	0.900	0.063
GrammarVAE	0.602	0.093	0.809	0.045
GraphVAE	0.557	0.670	0.616	0.261
GraphVAE/imp	0.562	0.52	0.758	0.179
GraphVAE NoGM	0.810	0.241	0.610	0.129
MolGAN	**0.981**	0.104	0.942	0.096
ARAE	0.862	0.935	0.371	0.299
**CRAG (QM9)**	0.872	**0.937**	**0.382**	**0.312**
**CRAG (ZINC)**	0.976	**0.971**	**1.000**	**0.948**

Bold values indicate the best performance w.r.t. the corresponding metric.

As expected, CRAG combines the advantages of the VAE-based methods and the GAN-based methods and improves the uniqueness and novelty of these models, but our model does not perform well in terms of validity. This is caused by the huge chemical space of the ZINC dataset, limiting the chances of producing new molecules. MolGAN, similar to our model, is trained based on the idea of adversarial attack. From Table 2, we can find that MolGAN shows high validity. This is because it sacrifices uniqueness, which means that a high value of a metric can be achieved by sacrificing other metrics. It is not advisable in the real task of *de novo* drug design. Therefore, we propose to use another metric: Novel/Sample (A×B×C), which is combined with validity (A), uniqueness (B), and novelty (C). This metric can be more suitable to evaluate the practicability of the generative model in real tasks. On the other hand, CRAG combines projected gradient descent to generate adversarial samples, which provides property information for adversarial samples and achieves cross-adversarial learning for CRAG. The model performance of CRAG is better than the ARAE-only (Hong et al., 2020) model.

In general, CRAG outperforms other models. The average effect of CRAG on the three metrics is expressed by Novel/Sample, which also shows that CRAG can be well applied on actual tasks.

#### 4.2.3 Performance of CRAG on Conditional Molecular Generation

In the field of *de novo* drug design, generation models are often required to generate related molecules based on specified molecular properties. In this section, we perform conditional molecular generation tasks based on the CRAG model, namely CCRAG (Conditional CRAG). In order to quantitatively compare the effectiveness of the generated molecules, we use the following three auxiliary indicators: logP, SAS, and TPSA. The water-octanol partition coefficient (logP) is defined as the ratio of a chemical’s concentration in the octanol phase to its concentration in the aqueous phase of a two-phase octanol/water system. Synthetics Accessibility Score (SAS) reflects the difficulty of synthesizing drug molecules. The score of SAS is between 1 (easy to make) and 10 (very difficult to make). Topological polar surface area (TPSA) estimates the polar surface area (PSA) of a compound from the bonding mode (topology) of the atoms in the molecule without considering the three-dimensional structure of the molecule. We simultaneously controlled the three properties of the molecule (logP, SAS, and TPSA), resulting in 10,000 molecules with given target properties. logP, SAS, and TPSA can be calculated by RDkit (Bento et al., 2020).

Table 3 summarizes the performance of CCRAG on the four indicators of validity, uniqueness, and novelty. CCRAG has a high success rate in conditional generation tasks, so CCRAG can easily control the generation of multi-property molecules under given fixed conditions.

**TABLE 3:** Performance of CRAG on Conditional Molecular Generation on the ZINC dataset, where the three conditions of logP, SAS, and TPSA are simultaneously controlled.

Condition	Validity (A)	Uniqueness (B)	Novelty (C)
(1.5, 2.0, 30)	0.913	0.937	0.999
(1.5, 2.0, 100)	0.834	0.992	0.999
(1.5, 5.0, 30)	0.892	0.995	1.000
(1.5, 5.0, 100)	0.603	1.000	1.000
(4.5, 2.0, 30)	0.894	0.998	1.000
(4.5, 2.0, 100)	0.826	1.000	1.000
(4.5, 5.0, 30)	0.579	1.000	1.000
(4.5, 5.0, 100)	0.273	1.000	1.000

#### 4.2.4 Property-Targeted Molecule Optimization

Optimizing a given molecule according to specific molecular properties is also one of the common tasks in *de novo* drug design. Here, we performed the quantification of drug-like properties (QED) (Bickerton et al., 2012) to the greatest extent given a single molecule. QED reflects the underlying distribution of molecular properties, which is intuitive, transparent, and straightforward to be implemented in many practical settings, and allows compounds to be ranked by their relative merit.

**FIGURE 6:**
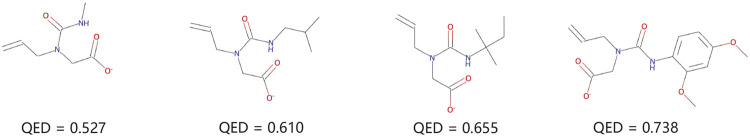
Chemical property optimization. Given the left-most molecule, we optimize the molecule in the direction of maximizing its QED property.

Figure 6 demonstrates a simple linear regression yields successful molecular optimization. We trained a linear regression model for molecular optimization with QED values. We selected a molecule with a low QED score and visualize the optimization process. The molecule can be optimized according to its gradient direction, so the molecule can obtain a greater increase in QED value with smaller possible changes.

## 5 Conclusion

In this paper, we propose a cross-adversarial learning method, named CRAG, for molecular generation using adversarial examples. Our model combines both the facticity of VAE-based methods and the diversity of GAN-based methods to further exploit the complex properties of Molecules. CRAG is based on a latent variable model to obtain the latent variables directly in GANs through adversarial training, rather than approximated by a predefined function. In adversarial training, CRAG uses continuous latent vectors instead of discrete molecular structures to avoid the difficulty of dealing with discrete variables. In addition, we also generate adversarial molecules through projected gradient descent to provide more property information and achieve cross-adversarial learning of CRAG. Extensively conducted on two benchmark datasets, which show the high uniqueness and high novelty of CRAG for molecular generation. Through the Novel/Sample metric, CRAG is validated to have a better overall effect, which indicates a promising sign that CRAG could become a new platform for AI-based molecular design in various chemical applications in the future.

## Data Availability

The original contributions presented in the study are included in the article/Supplementary Material, further inquiries can be directed to the corresponding author.
